# *In vitro* isolation and identification of entomopathogenic fungus (*Metarhizium pinghaense*) and assessment of its virulence against whiteflies and aphids

**DOI:** 10.1371/journal.pone.0338072

**Published:** 2026-07-22

**Authors:** Haiyan Hu, Yali Wang, Chunyan Li, Ranran Zhang, Fangyu Liu, Xiaoan Sun

**Affiliations:** Shandong Provincial Facility Horticulture Bioengineering Research Center, Weifang University of Science and Technology, Shouguang, Shandong, China; National Institute of Agricultural Research - INRA, MOROCCO

## Abstract

*Bemisia tabaci* (Gennadius) and *Aphis gossypii* (Glover) are two notorious pests of vegetables grown in greenhouses and their outbreaks can be devastating and cause a significant loss in yield and quality. The prevail and effective control of the pests mainly relies on frequent applications of pesticides, which is likely to negatively impact environments and human health through residue, resurgence, and resistance. To avoid negative “3R” issues brought forth by chemicals, entomopathogenic fungus such as *Metarhizium* sp. are widely used as biocontrol agents even if they are different in their population, adaptability and pathogenicity among various species and isolates collected from different insect hosts and environments. To screen for the entomopathogenic fungi with optimal pathogenicity against the piercing-sucking pests *B. tabaci* and *A. gossypii*, we have isolated, purified four strains (SG-A, SG-B, SG-C, and SG-D) of *Metarhizium* sp. using yellow mealworms (*Tenebrio molitor*) as the bait, and identified them as *Metarhizium pinghaense* morphologically and molecularly. Bioassay results have indicated that four isolates of *M. pingshaense* infected both *B. tabaci* and *A. gossypii* with some differences in the virulence and median lethal time. Both *B. tabaci* and *A. gossypii* nymphs showed an increase in the mortality rate as the spore concentration rose. Four *M. pingshaense* strains also exhibited the different pathogenicity against *B. tabaci* and *A. gossypii* at various levels: At an inoculum concentration of 1 × 10^8^ conidia/mL, the cumulative corrected mortality of *B. tabaci* nymphs 8 days post-treatment ranked in the order: SG‑A > SG‑C > SG‑B = SG‑D. For *A. gossypii*, the cumulative corrected mortality followed the order: SG‑A > SG‑B > SG‑D > SG‑C.Strain SG-A demonstrated the maximized pathogenicity against nymphs of *B. tabaci* and *A. gossypii* 8 days after the treatment (LC_50_ = 7.00 × 10^4^ and 4.21 × 10^5^ conidia/mL, respectively). At the spore concentration at 1 × 10^8^ conidia/mL, its cumulatively corrected mortality reached 94.44% and 96.67% (LT_50_ = 4.13 d and 2.61 d, respectively). Strain SG-C showed an optimal pathogenicity against *B. tabaci* nymphs only with a cumulative corrected mortality of 87.78% and an LT_50_ at 4.30 d 8 days after the treatment. In contrast, strain SG-B exhibited relatively high pathogenicity against *A. gossypii* nymphs only with a cumulative corrected mortality of 72.22% and an LT_50_ at 2.31 d 8 days after the treatment. Therefore, *M. pinghaense* SG-A should be a potential biocontrol agent to manage whiteflies and aphids at their nymph stage during the vegetable production season.

## Introduction

Whiteflies *Bemisia tabaci* (Gennadius) (Hemiptera: Aleyrodidae) and aphids *Aphis gossypii* (Glover) (Hemiptera: Aphididae) are important pests on over 1000 and 700 various plant hosts (such as cucumbers, peppers, eggplants, tomatoes, etc.) and their host ranges are cross-overlapping, causing serious damage under suitable temperature conditions and humidity throughout the year [1–2]. Moreover, as the polyphagous and sucking pests, they feed on a wide range of plants, affect the vegetable growth and development through feeding on plant hosts and spreading more than 100 viruses such as watermelon mosaic virus (WMV), tomato yellow leaf curl virus (TYLCV), and tomato chlorosis virus (ToCV), and devastate the crop and vegetable production [3–6].

For last several decades, chemical control of such insect pests is effective in mitigating crop loss through pest prevention and frequent applications of pesticides [7–8]. However, a long-term dependence on chemical pesticides has carried a series of problems such as environmental pollution, plant resistance to insecticides (e.g., pyrethroids, organophosphates, and neonicotinoids), and pesticide residues [9–11]. Therefore, it is urgent to find a green and efficient approach to reduce the use of pesticides. At present, biological controls utilizing the microbial parasitism or antagonism have gradually gained a great attention for their widely accepted applications in preventing and controlling of pests in facility-grown vegetables. Entomopathogenic fungi (EPF), especially *Metarhizium* spp., are currently one of the most widely used biocontrol fungi. Their application can help supplement or reduce the use of chemical pesticides on crops. Of the 171 commercially available insect pathogenic fungi worldwide, approximately 34% of them belong to the genus *Metarhizium* [12]. The infection process of *Metarhizium* spp. is complex, and can be divided into five stages: Recognition and attachment of conidia to the host insect skin, germination and formation of penetrating pegs to invade hemocoel, establishment and killing of the insect, and production of a large number of conidia [13–14]. *Metarhizium* spp. prove to be effective on a variety of insect pests, such as African sugarcane longhorn beetle (*Cacosceles newmannii* (Coleoptera: Cerambycidae)) [15], bagrada bug (*Bagrada hilaris* (Hemiptera: Pentatomidae)) [16], and red palm weevil (*Rhynchophorus ferrugineus* (Coleoptera: Curculionidae)) [17–18]. However, environmental parameters, especially high temperature and humidity, greatly enhance the pathogenicity of those marketed *Metarhizium* spp. to infect and colonize their host insects [19], which makes *Metarhizium* more feasible and effective in suppressing the population of both whiteflies and aphids in facility vegetable productions.

*Metarhizium* spp. is one of the most intensively studied and commercially valuable genera of entomopathogenic fungi, which are commonly isolated from soil environments [20–21]. To date, more than 80 valid species of *Metarhizium* have been formally described, among which *Metarhizium anisopliae* is one of the most widely utilized species in the genus [20–23]. However, there are few reports on the pathogenicity of *Metarhizium* spp. to sucking insect pests such as whiteflies and aphids, so screening for more virulent strains of wild-type *Metarhizium* spp. for their potential application as biocontrol agents becomes much necessary to mitigate the threat imposed by *B. tabaci* and *A. gossypii*. With this study, we intended to use four purified *Metarhizium* isolates obtained from soil samples in different regions for their identity by morphological and molecular analyses and to evaluated their pathogenicity against both *B. tabaci* and *A. gossypii*, which should provide sufficient first-hand information on the biological control of sucking insect pests for the facility’s vegetable production.

## Materials and methods

### Soil sampling sites

Four fungal isolates were obtained from soil samples collected from vegetable-growing greenhouses located at the Shouguang Modern Agriculture Center and Seed R&D Station with Weifang University of Science and Technology, Shouguang, Shandong Province (36°53'00.00'N, 118°42'00.00'E), China.

### Fungal isolation

Five soil samples were collected from four plots planted with sweet potato, Chinese cabbage, cucumber, and tomato, respectively at 4 directions (N, S. E and W) and in the center of each plot, 3–5 replicates per treatment. Prior to the sample collection, litters, weeds, rocks and other debris on the soil surface were removed to avoid contamination. 20 cm deep soil (~500 g) was dug out vertically with a sterile shovel, placed and sealed into sterile bags. Tools were disinfected with alcohol between different samplings to prevent cross-contamination. The sterile bags were labeled with information including sequential number, location, depth, date, habitat, treatment, and other relevant information. After being transported back to the laboratory, sample soils were used to isolate entomopathogenic fungi using nymphs of yellow mealworm, *Tenebrio molitor* (Coleoptera: Tenebrionidae) as the baits [24].

Soil samples were poured onto a piece of clean newspaper, spread, and placed in a cool, well-ventilated, and dry area of the laboratory (room temperature 20–25 °C, relative humidity 30–40%) air-dried for 24 hours. 180 g of each soil sample was placed into a 250 mL sterilized flask spray the appropriate amount of distilled water. Ten of the 6th-7th instar (1.2–1.8 cm long) healthy yellow mealworm nymphs were placed in each sterilized flask on the sample soil and kept in an artificial phenological incubator (spx) at (25 ± 2) ℃, RH 70% ± 5%, and photoperiod 12L:12D. All samples kept in the bottle were moisturize with an appropriate water spray and turned upside down at least 3 times a day without food supply throughout the incubation. The infected mealworms were removed from the soil and placed in a sterile 24-well plastic culture plate for further cultivation. The infected mealworm larvae were cut into small pieces, disinfected with 75% alcohol for 3–5 min, washed with sterile ddH_2_O for 5 times, and then placed onto SDAY nutrient plates (3 pieces of larvae per plate) containing 0.3 g/L tetracycline and 0.3 g/L kanamycin) for 10–14 days at 26 ℃. Fungal colonies were picked after 3–5 d growth and a single colony was obtained through transferring the edge of fungal colonies several times [25–27].

### Identification of fungal

#### Morphological identification.

A slide culture method was used by transferring a 5 × 5 mm PDA plug onto a sterilized slide and placing a small amount of purified fungal hyphae on the plug. The inoculated slide was covered and placed in a large Petri dish with a small amount of water in it, kept at 26 ℃ for 3 days and observed daily. The morphological characteristics of mycelia, conidiophores and conidia were observed under the microscope and photographed with the Motic Images Plus 2.0 system. The species were identified according to the morphological characteristics of the colony, mycelium, spore, and spore stalk [25,28].

#### Molecular phylogenetic analysis of fungi.

The total DNA of fungal isolates cultivated on PDA for 7 days was extracted by Norgen Biotek Fungi DNA Isolation Kit and the rDNA-ITS sequence of the strain was amplified by PCR using the universal primers ITS4/ITS1F. Also, Pbeta-F/Pbeta-R and PRPB2-F/PRPB2-R primers were used to amplify β-tubulin and RPB2, respectively (Table 1). The PCR reaction systems of DNA-ITS sequence, β-tubulin sequence and RPB2 sequence are: upstream and downstream primers (10mmoL) 1 μL each, PCR Mastermix was 12.5 μL, template DNA 1 μL, ddH_2_O 9.5 μL, a total of 25 μL.

**Table 1:** Primers used to identify the fungal isolates.

Primer	Sequence
ITS4	5′-TCCTCCGCTTATTGATATGC-3′
ITS1F	5′-CTTGGTCATTTAGAGGAAGTAA-3′
Pbeta-F	5′-CCCTCCATTGTCTAGGACC-3′
Pbeta-R	5′-CACATCATTGACGGGACTTAC-3′
PRPB2-F	5′-TTGTCCAATTATTTGCGAAGAT-3′
PRPB2-R	5′-CGCAGCAGTTCAGATACAGAGT-3′

The PCR reaction procedures of the three sequences were: Pre-denaturation at 95 ℃ for 3 min, denaturation at 94 ℃ for 1 min, annealing at 55 ℃ for 1 min, extension at 72 ℃ for 1.5 min, 35 cycles. 1% agarose gel electrophoresis was performed and the amplification results were observed under the gel imaging system. PCR products were sent to Beijing Liuhe Bada Gene Technology Co., Ltd., Beijing, China for sequencing.

The sequences of rDNA-ITS, β-tubulin, and RPB2 were sequenced by the Blast program (http://blast.ncbi.nlm.nih.gov/Blast.cgi) and gene sequences in GenBank for homology analysis. The corresponding sequences of common related species were downloaded, and the corresponding sequences of *Beauveria bassiana* were taken as exogenous species. MEGA4.0 software ClustalX method was used to compare multiple sequences, and molecular phylogenetic trees were constructed by the neighbor-joining method. The system tree was tested with Bootstrap and repeated 1000 times.

### Insect rearing

*B. tabaci* and *A. gossypii* adults were collected on pepper and cucumber plants, respectively from a greenhouse with the R&D station and raised on their host seedlings. The population of whitefly was identified as *B. tabaci* Q biotype based on mitochondrial DNA COⅠ gene. After being reared for 5 generations, the 4th instar nymphs of both insects at the same age were used for the further experiments.

### Virulence bioassays

#### Preparation of conidial suspension.

Four fungal isolates were grown on PDA plates for 14 days and the conidial spores were gently scraped off each of the colonies with soft-tipped sterilized spatula. Conidial spores were then rinsed once in a 200 ml beaker with 0.05% polyoxyethylene sorbitan monooleate solution (Tween 80, Sigma–Aldrich), resuspended in distilled water and mixed fully on a vortex shaker for 10 min. The average concentration of the spore suspension was determined by hemocytometer under the microscope for 5 times. Five concentrations (1 × 10^4^, 1 × 10^5^, 1 × 10^6^, 1 × 10^7^ and 1 × 10^8^ conidia/mL) of fungal spore suspension were prepared for further use. To determine the median lethal concentration of each fungal isolate to kill 50% of target insects hosts, (LC_50_), the suspension (1 × 10^8^ conidia/mL) was used to calculate the median killing time to kill 50% of the target insects (LT_50_).

### Insect bioassay

#### Laboratory bioassay of four fungi isolates against *B. tabaci* and *A. gossypii.*

The bioassay was carried out following the method described by Paradza *et al.* (2021) [29] with some modification. For each treatment, 30 4th-instar nymphs of *B. tabaci* or *A. gossypii* were placed onto a pepper leaf disc (3 cm in diameter) using a fine brush. Leaf discs were selected from leaves at the same leaf position to ensure a consistent phytochemical background and eliminate variation among treatment groups. The leaf discs carrying nymphs were placed in Petri dishes containing 10 mL of 1% agar medium. A 2 mL aliquot of fungal conidial suspension at each concentration (1 × 10^4^, 1 × 10^5^, 1 × 10^6^, 1 × 10^7^, and 1 × 10^8^ conidia/mL) was applied using a small handheld sprayer, ensuring thorough coverage of all nymphs. After air-drying, the leaf discs were inverted and incubated at 25 ± 1 °C, 70 ± 5% RH, under a 12 h light: 12 h dark photoperiod for 8 days. Throughout the incubation, the infection status of the parasitized nymphs of whiteflies or aphids was observed daily and the hyphal growth of entomopathogenic fungus and the fungal sporulation were recorded. A sterile water containing only Tween-80 was sprayed as a control, and the mortality rate of the control was kept below 10%. All treatments were replicated 3 times.

### Data analysis

The mortality rates were corrected using Abbott’s formula [30]. One-way analysis of variance (one-way ANOVA) was performed on the mortality data using SPSS 26.0 software (IBM Corp., Armonk, NY, USA), and Duncan’s new multiple range test was conducted for post-hoc pairwise multiple comparisons. The Probit regression procedure was used to calculate the slope of the regress curve, intercept, 95% confidence limits, the lethal concentrations of 50% (LC_50_), and spore concentration of 1 × 10^8^ conidia/mL of median lethal time (LT_50_) of the isolates by SPSS 26.0 software, and calculate the regression equation based on the slope of the regress curve and intercept.

## Results

### Species identification of *Metarhizium* spp

#### Morphological identification.

**Fig 1:**
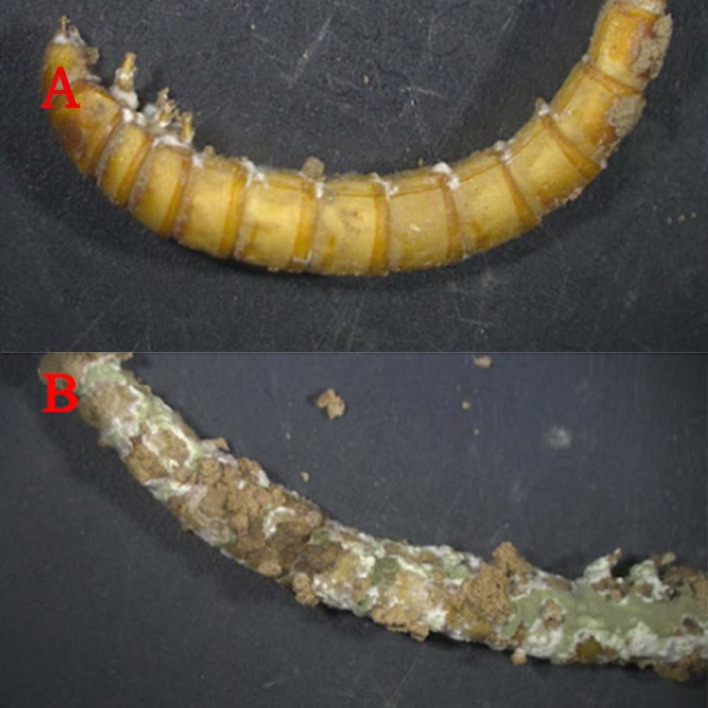
Appearance of yellow mealworms infected and colonized by *Metarhizium* spp. in two (A) and four (B) days.

**Fig 2:**
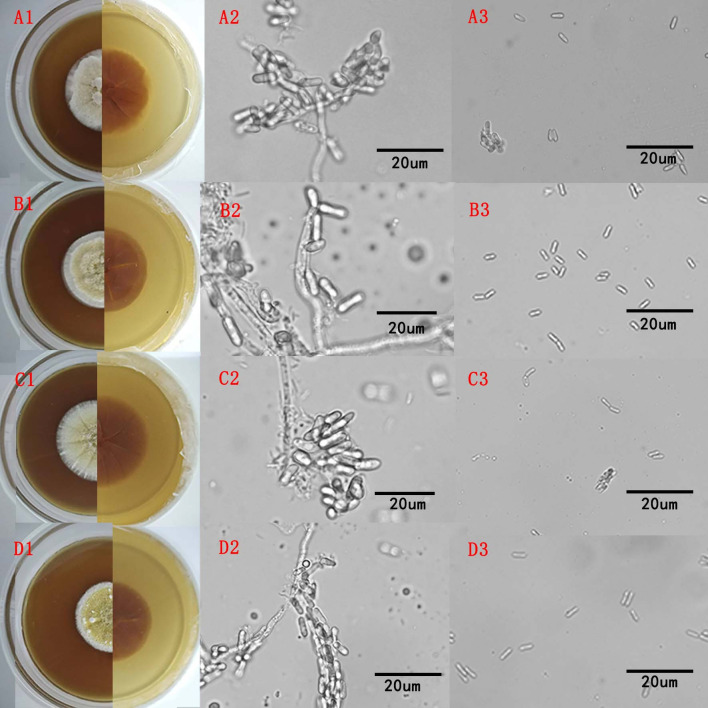
Morphological characteristics of *Metarhizium* sp. isolates (A-1, B-1, C-1, D-1), colonies of *Metarhizium* cultured on SDAY at 25℃ for 14 days (A-2, B-2, C-2, D-2), and sporophore and conidial morphology of each isolate (A-3, B-3, C-3, D-3), respectively.

**Fig 3:**
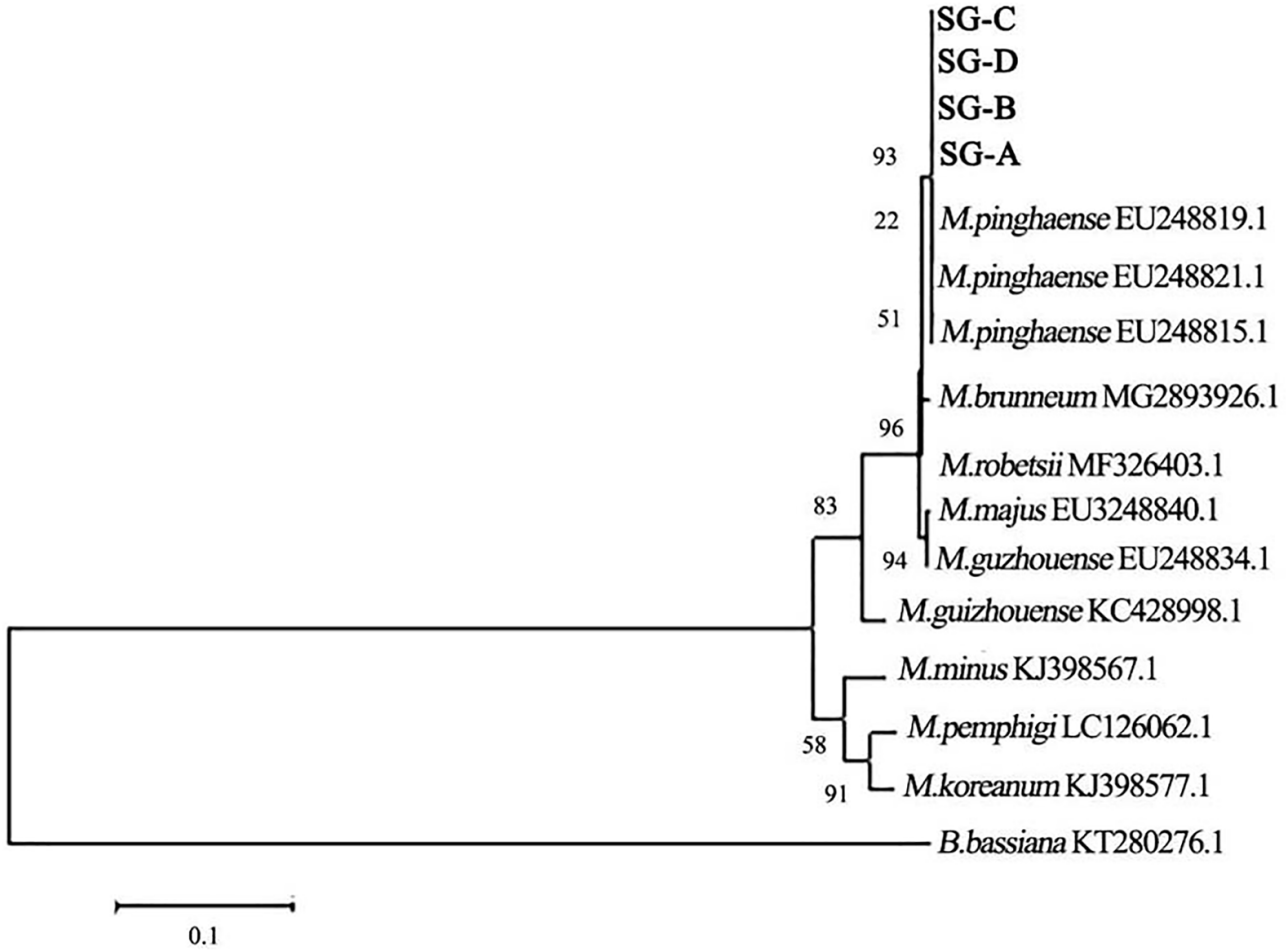
The phylogenetic tree of four isolates compared to other related *Metarhizium* species based on their sequence of the beta-tubulin coding gene through the neighbor-joining method.

Four *Metarhizium* spp. isolates were collected from soil 20 cm below the surface at different locations of the research center. The isolation of insect-parasitic fungi from the soil was carried out using yellow mealworms as the bait. After infecting the yellow mealworms, white mycelia appeared first in the intersegment portions of the worm body and then dark-green fungal spores gradually showed up (Fig 1A-B). The appearance of fungal colony on the reverse side of SDAY Petri dish plates was reddish-brown, and folded longitudinally (Fig 2, A-1, B-1, C-1, D-1). Spores were yellow-green and oval, 2.01–2.58 × 5.84–6.25 µm (Fig 2, A-2, B-2, C-2, and D-2). Under the microscope, hyphae were septate and the conidial peduncles were like mycelia with single or multiple small peduncles at the top, which were densely and neatly formed, and long chain-like conidia were formed at the end (Fig 2, A-3, B-3, C-3, D-3).

#### Molecular biological identification.

**Fig 4:**
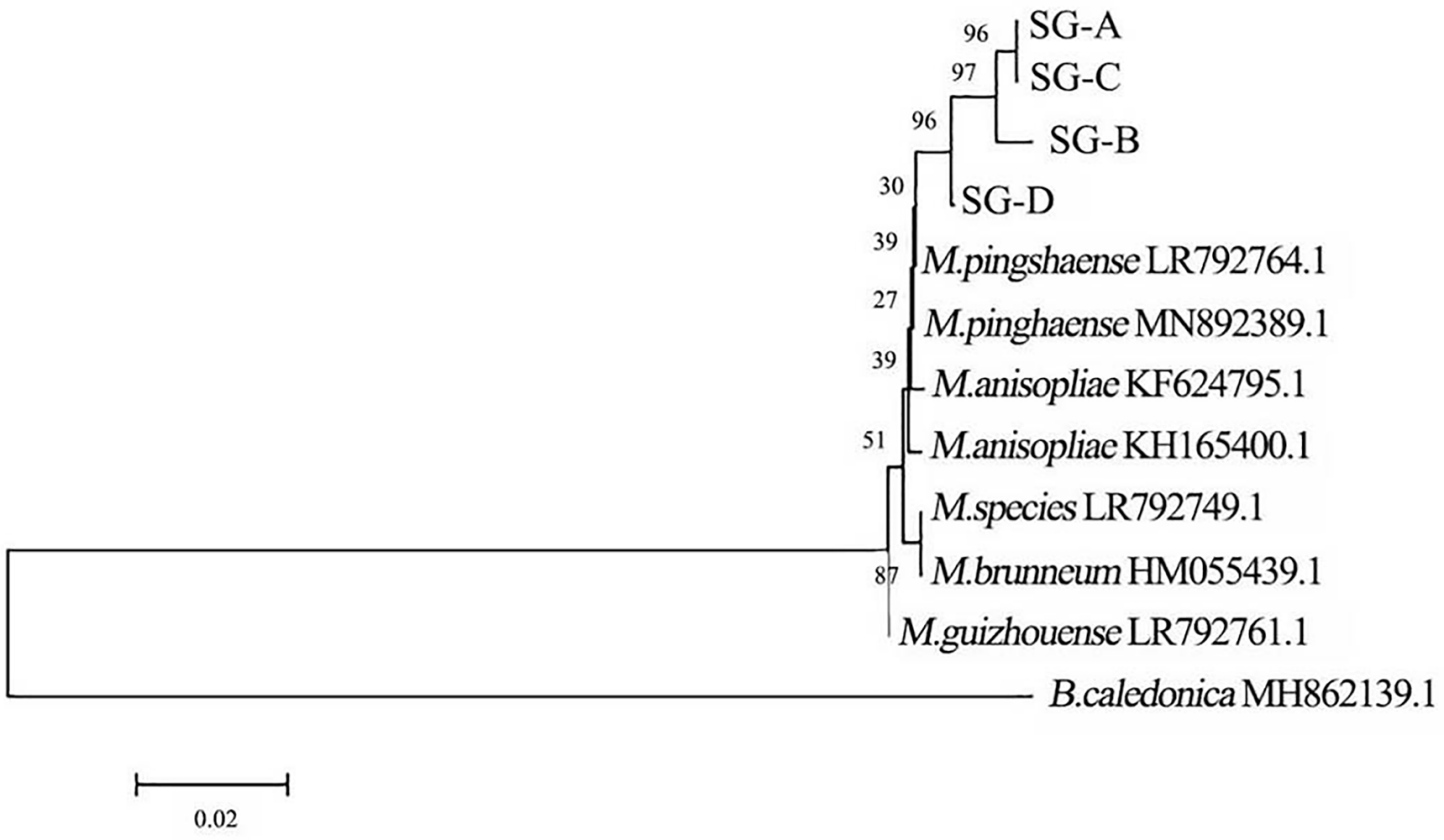
The phylogenetic tree of four isolates compared to other related *Metarhizium* species based on their sequence of the rDNA-ITS coding gene through the neighbor-joining method sequence.

**Fig 5:**
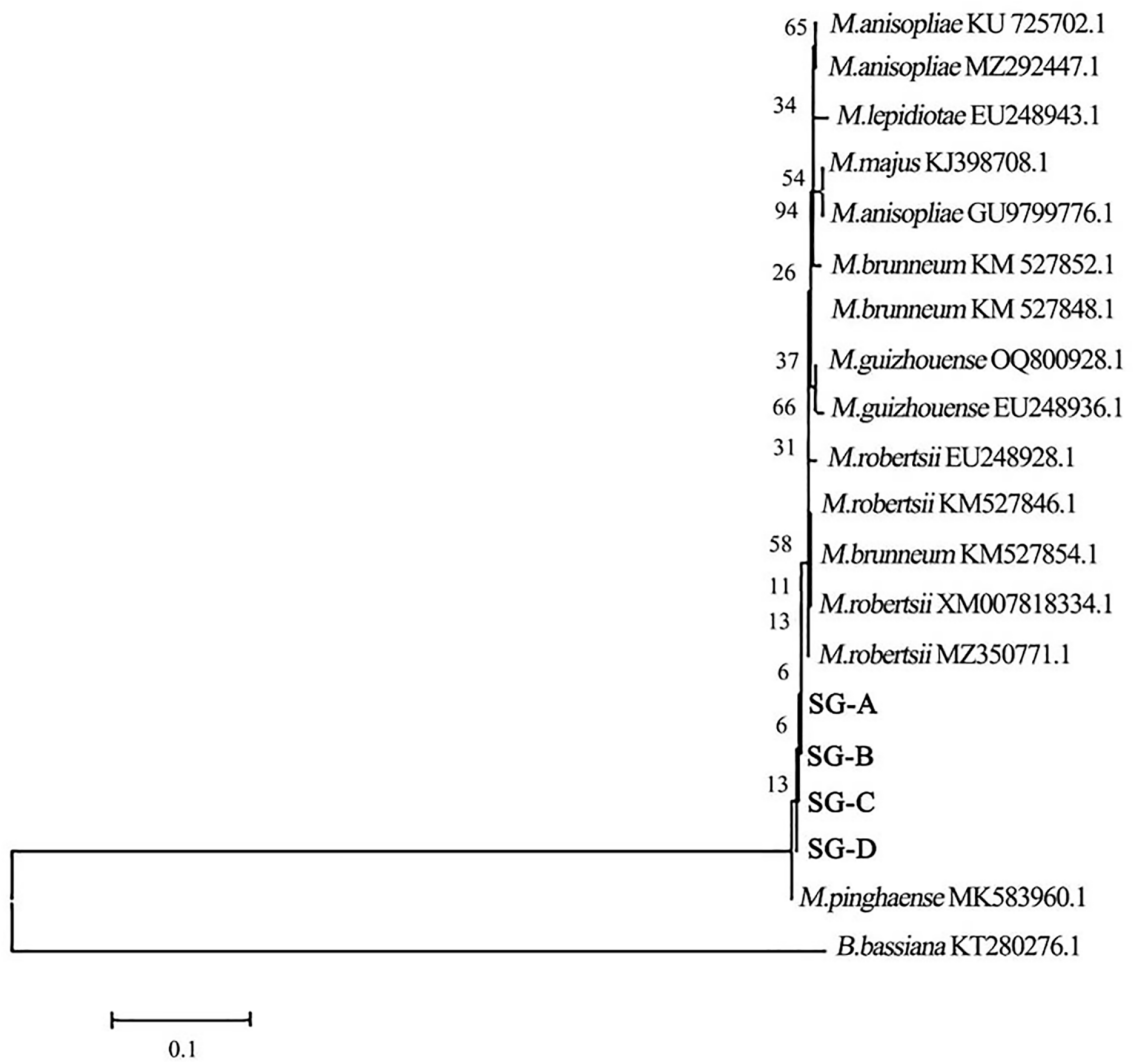
The phylogenetic tree of four isolates compared to other related *Metarhizium* species based on their sequence of the RPB2 coding gene through the neighbor-joining method.

rDNA-ITS, β-tubulin, and RPB2 sequences were amplified with the DNA extracted from four *Metarhizium* isolates and the sequence alignment of 559 bp, 1246 bp, and 969 bp fragments were respectively analyzed in GenBank by BLAST, showing a 99% genetic similarity with *Metarhizium pinghaense* in the constructed phylogenetic tree in comparison with the sequences of other *Metarhizium* species (Fig 3–5). Therefore, all of four isolates were identified as *M. pinghaense* according to their morphological traits and molecular identification.

#### Bioassay.

**Fig 6:**
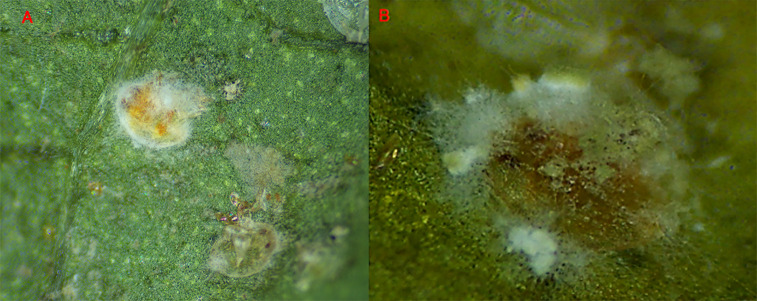
Infected fourth instar nymphs of *B. tabaci* showing (A) proliferation of hyphae on the surface of the insect (2 d post-inoculation), and (B) massive sporulation on the insect corpses (4 d post-inoculation).

Two days after being submerged in the *M. pinghaense* spore suspension, the skin of the 4th instar whitefly nymphs turned dark with fluffy white fungal hypha (Fig 6A). Four days after inoculation, the body of inoculated *M. pinghaense* nymph appeared to be yellow-green with heavy sporulating bodies (Fig 6B).

**Fig 7:**
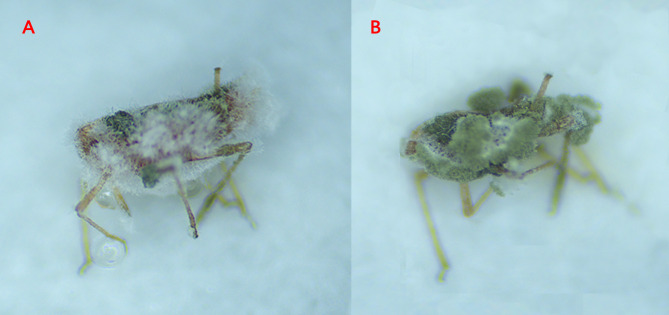
Infected fourth instar nymphs of *A. gossypii* showing (A) proliferation of hypha on the surface of the insect (2 d post-inoculation), and (B) massive sporulation on the insect corpses (4 d post-inoculation).

Two days after being submerged in the *M. pinghaense* spore suspension, the skin of the 4th instar *A. gossypii* nymphs turned dark brown with light fungal mycelia (Fig 7A). Four days after inoculation, the body of the inoculated *M. pinghaense* nymph appeared to be black with heavy spore mass. The dead nymph bodies were covered with massive chartreuse-spores (Fig 7B).

**Fig 8:**
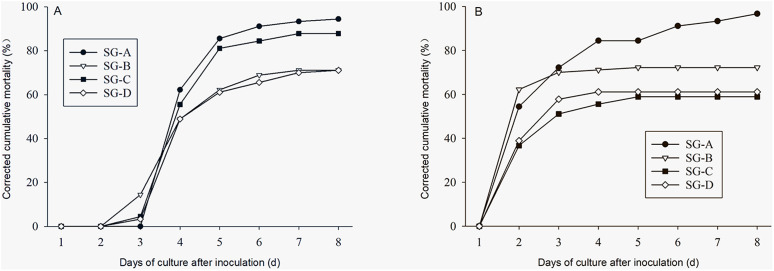
The mortality of the 4th instar nymphs of *B. tabaci* (A) and *A. gossypii* (B) over time after inoculation with four different *M. pinghaense* isolates at 1 × 10^8^ conidia/ml. Experiments were performed in triplicate.

The 4th instar nymphs of whitefly inoculated 1 × 10^8^ conidia suspension of four *M. pinghaense* isolates started to die on Day 2 and the mortality increased exponentially through Day 5 (Fig 8). The *M. pinghaense* SG-A isolate scored the highest percentage causing whitefly nymphs to die (94.44% mortality in 8 days), followed by the SG-C, SG-B, and SG-D isolates (79.90% mortality in 8 days), respectively (Fig 8A). Similarly, all 4 *M. pinghaense* isolates were lethal to *A. gossypii* nymphs 2 days after inoculation. The SG-A isolate showed the highest mortality (96.67% in 8 days), followed by SG-B, SD-D, and SG-C (58.89% in 8 days) (Fig 8B).

There were differences in the mortality rates of *B. tabaci* and *A. gossypii* 8 days after infection by four strains of *M. pingshaense* at a spore concentration of 1 × 10^8^ conidia/ml. Among them, the SG-A strain exhibited the highest pathogenicity against both *B. tabaci* and *A. gossypii* (Table 2). After 8 days of treatment, the mortality rates of *B. tabaci* and *A. gossypii* nymphs were 94.44% and 93.33% respectively, showing significant differences from those of other strains (Table 2). In contrast, the pathogenicity of SG-C and SG-D strains against *B. tabaci* nymphs was higher than that against *A. gossypii* nymphs (Table 2). The SG-B strain showed comparable pathogenicity against both *B. tabaci* nymphs and *A. gossypii* nymphs (Table 2). There were differences in the median lethal time (LT_50_) of four *M. pinghaense* isolates against the 4th instar *B. tabaci* nymphs treated with 1 × 10^8^ conidia/ml spore suspension. *M. pinghaense* SG-A only took 4.13 days on average to kill the wihtefly nymphs while the isolate SG-D needed 5.09 days on average to cause the death (Table 2). In terms of LT_50_ of four *M. pinghaense* isolates, SG-B took 2.31 days on average to kill its target nymphs, SG-C required at least 4.27 days on average to be lethal against *A. gossypii* nymphs at 1 × 10^8^ conidia/ml (Table 2).

**Table 2:** Cumulative mortality and median lethal time (LT_50_) after 8 days of treatment with spore suspension of four *M. pinghaense* isolates at 1 × 10^8^ conidia/ml.

Isolate	% Mortality of *B. tabaci* (Mean ± SE)	LT_50_ (days) 95% FL	% Mortality of *A. gossypii* (Mean ± SE)	LT_50_ (days) 95% FL
SG-A	94.44 ± 5.09a	4.13(3.331-4.786)	93.33 ± 3.34a	2.61(0.572-4.208)
SG-B	71.11 ± 6.94b	4.87(4.377-5.415)	72.22 ± 5.09b	2.31(1.927-2.659)
SG-C	87.78 ± 5.09a	4.30(3.515-4.988)	58.89 ± 7.70c	4.27(2.842-6.704)
SG-D	71.11 ± 5.09b	5.09(4.168-6.201)	61.11 ± 3.85c	3.85(2.238-6.185)

Note: Means within the same column followed by the same letter do not differ significantly at P < 0.05 (Tukey’s HSD). FL represents the 95% fiducial limits. Error bars = ±SE.

The virulence of four *M. pinghaense* isolates against *B. tabaci* and *A. gossypii* nymphs were quite different (Table 3). Among them, isolate SG-A exhibited the strongest virulence against *B. tabaci* nymphs, with an LC₅₀ value of 7.00 × 10⁴ conidia/mL, followed by isolates SG-C and SG-B, while isolate SG-D displayed the lowest virulence. The isolate SG-A exhibited the highest virulence against *A. gossypii* nymphs, with an LC_50_ value of 4.21 × 10^5^ conidia/mL, followed by isolates SG-B and SG-D, while isolate SG-C showed the lowest virulence (Table 3).

**Table 3:** The virulence of four *M. pinghaense* isolates against the 4th instar nymphs of *B. tabaci* and *A*. *gossypii.*

Isolate	Insect species	Regression equation	LC_50_ values（conidia/ml）	χ² value	95% confidence interval	P value
SG-A	*B. tabaci*	Y = −0.311 ＋ 0.368X	7.00 × 10^4^	2.725	0.64 × 10^4^-2.81 × 10^5^	0.436
	*A*. gossypii	Y = −0.646 ＋ 0.398X	4.21 × 10^5^	0.58	9.38 × 10^4^-1.47 × 10^6^	0.901
SG-B	*B. tabaci*	Y = −0.451 ＋ 0.211X	1.36 × 10^6^	1.325	8.17 × 10^4^-3.99 × 10^7^	0.723
	*A*. gossypii	Y = −0.469 ＋ 0.215X	1.54 × 10^6^	0.201	1.07 × 10^5^-4.61 × 10^7^	0.977
SG-C	*B. tabaci*	Y = −0.348 ＋ 0.338X	1.37 × 10^5^	1.858	1.34 × 10^4^-5.80 × 10^5^	0.602
	*A*. gossypii	Y = −0.978 ＋ 0.306X	1.58 × 10^7^	0.105	3.06 × 10^6^-4.21 × 10^8^	0.991
SG-D	*B. tabaci*	Y = −0.469 ＋ 0.215X	1.54 × 10^6^	1.176	1.07 × 10^5^-4.61 × 10^7^	0.759
	*A*. gossypii	Y = −0.848 ＋ 0.278X	1.12 × 10^7^	0.094	1.95 × 10^6^-3.91 × 10^8^	0.993

## Discussion

The long-term and extensive use of chemical insecticides has raised serious issues such as pest resistance, food safety, environmental pollution, and health [1,10]. Therefore, it is imperative to apply the Integrated Pest Management (IPM) concept to minimize the amount and frequency of chemical pesticides and improve their efficacy as a safe and effective strategy in plant pest control. Biological control, especially through using parasitic fungi to control pests has been widely used. *Metarhizium* sp. is one of the most studied and wildly used biological control agents that can be easily isolated from the rhizosphere and has a potential to be used in the field after initial screening and evaluation with the advantages of being safe and adaptive in the facility environment [31].

As the first step in the researches for ideal *Metarhizium* species as biocontrol agents, isolation and identification of four entomopathogenic *Metarhizium* isolates as *M. pingshaense* were successful using yellow mealworms as the baits with this study. Although the morphological characteristics such as colony appearance, spore size, and spore shape of the *Metarhizium* isolates on SDAY plats (Fig 2) are consistent with each other and to those described in previous studies [22,32], the confirmatory assurance of fungal speciation rely on the molecular diagnosis, especially the alignment of key gene sequences [31]. The constructed phylogenetic tree of partial sequences of the Pbeta (Fig 3), ITS (Fig 4), and PRPB2 genes (Fig 5) of 4 *Metarhizium* isolates in comparison with those sequences of other *Metarhizium* spp. deposited in GeneBank has demonstrates that four isolates were grouped in a small evolutionary branch with a highest homology with *M. pingshaense* reported earlier, providing a solid evidence to support our identification and speciation of all four strains. The relatively low bootstrap support values (below 50%) at several key nodes of the phylogenetic trees presented in Figs 3–5 of this study may be mainly attributed to the rapid radiation diversification of the *Metarhizium* species complex during the Pleistocene. The compressed speciation timeframe resulted in exceedingly low sequence divergence among closely related species, and thus insufficient parsimony informative sites within the selected barcode genes (ITS, TEF1-α, and RPB2). Furthermore, the widespread incomplete lineage sorting (ILS) and interspecific gene introgression events prevalent in this complex also give rise to conflicting phylogenetic signals, which represent the primary drivers of low bootstrap support for terminal clades. This phenomenon is ubiquitous in phylogenetic analyses of *Metarhizium* species and has been extensively documented in previously published studies [33].

Early studies on the pathogenicity of *M. pingshaense* other other insect pests suggest it is able to infect many Lepidopteran species such as rice leaf folder (*Cnaphalocrocis medinalis*) [23] and yellow peach moth (*Conogethes punctiferalis*) [31], and Coleopteran ones such as longhorn beetles (*Cacosceles newmannii*) [15] and red palm weevil (*Rhynchophorus ferrugineus*) [22] with an acceptable efficacy. However, there are still few reports on the assessment of the *M. pingshaense* pathogenicity against insect pests with the piercing or sucking mouthparts, especially against aphids and whiteflies. Previous researches also indicate that different isolates of a entomopathogenic fungus vary in their infective ability to the host pests [17]. Our laboratory results derived from the virulence bioassays showed that the four *M. pingshaense* strains were all pathogenic to the 4^th^ instar nymphs of *B. tabaci* and *A. gossypii*. However, the four *M. pinghaense* strains differed significantly in virulence against both *B. tabaci* and *A. gossypii* nymphs, with significant virulence variation observed even within the same host species. This may be explained by genetic differentiation and sequence polymorphisms in virulence-related genes among strains. Consistently, *M. pinghaense* isolates from Vietnam and Italy showed comparable virulence to *R. ferrugineus* but distinct enzymatic activity and toxin profiles, further supporting the regulatory role of genetic lineages on virulence phenotypes [34].The mortality rates of four *M. pingshaense* isolates against *B. tabaci* and *A. gossypii* ranged from 71.1–94.4% and 58.9–96.7% at a concentration of 1 × 10^8^ conidia/mL. However, the level of their virulence based on mortality, median lethal concentration (LC_50_) and median lethal time (LT_50_) values differed among the isolates, indicating that the SG-A is the most virulent strain to infect the 4th instar nympha of *B. tabaci* and *A. gossypii* (LC_50_ = 7.00 × 10^4^ and 4.21 × 10^5^ conidia/mL, respectively, Table 3). The reason that more SG-A spores are needed to kill the same number of whiteflies (*B. tabaci*) may be due to the bigger sizes of aphid (*A. gossypii*) bodies, more SG-A conidial spores are required to land on smaller insect bodies. Similar studies have shown that the isolates of *M. anisopliae* AAUDM-43 have high pathogenicity against tobacco whitefly nymphs with an LC_50_ at 5.4 × 10^4^ conidia/ml, respectively [35]. Norhelina’s research also demonstrates that the isolate GJ4 of *M. anisopliae* has the highest pathogenicity against tobacco whiteflies, with an LD_50_ at 6.62 × 10^4^ conidia/ml [36]. The virulence levels reported in two previous published studies were highly consistent with those obtained in our study, indicating that the SG-A strain has considerable application potential for the control of *B. tabaci* nymphs. Enoh *et al*. [37] reported that the *Metarhizium anisopliae* strain MIITAC6.2.2 exhibited high pathogenicity against the banana aphid *Pentalonia nigronervosa*, with a median lethal concentration (LC_50_) of 3.12 × 10^2^ conidia/mL at 10 days post-inoculation. The virulence of this strain was higher than that of the strains tested in our study, which may be attributed to the differences in fungal strains and target pest species between the two studies.

In this study, the relatively wide 95% confidence intervals observed in the virulence regression equation of strain SG-B against *B. tabaci* may be attributed to natural variation among test insects, minor differences in inoculation uniformity, and limited sample sizes in each treatment. Such variability is common in bioassays involving entomopathogenic fungi and hemipteran pests, as individual susceptibility to fungal infection can vary even among nymphs of the same instar. Although the confidence intervals are relatively broad, the overall mortality trends among different fungal strains remain consistent and comparable with previous studies, indicating that the main conclusions are reliable.

The median lethal time (LT_50_) value of different isolates against *B. tabaci* and *A. gossypii* are different under the same concentration (1 × 10^8^ conidia/mL, Table 2). The LT_50_ values of four *M. pingshaense* isolates against *A. gossypii* takes all shorter time than that of those against *B. tabaci*, possibly due to the fact that aphids obtain more fungal spores on their appendages and body cavities through the crawling and are more susceptible to the fungal infections [37]. Another possible explanation is that the 4th-instar nymphs of *A. gossypii* have a larger body size than those of *B. tabaci*, and their honeydew secretion is consistently higher than that of *B. tabaci*. Higher honeydew production is more conducive to the infection and colonization of *M. pinghaense*, thereby shortening the infection period. Furthermore, differences in the integumental cuticle thickness between *A. gossypii* and *B. tabaci* nymphs may also account for the variation in the infection time of *M. pinghaens*e conidia [38]. The SG-A isolate takes less time to kill *B. tabaci* with an LT_50_ at 4.13 d, while the SG-B does that on *A. gossypii* with LT_50_ at 2.31 d.

Although the *Metarhizium pinghaense* strain SG-A in this test has strong pathogenicity to nymphs of *B. tabaci* and *A. gossypii*, it is only limited to indoor virulence determination, and its field control effect is still uncertain. In addition, this study only measured the virulence of the strain against 4th-instar nymphs, and the lethal effect on other insect stages is still unknown. In the follow-up work, we will continue to explore a series of issues including the virulence of the strain against nymphs at other instars, optimization of solid-liquid biphasic fermentation conditions of *M. pinghaense* bacterial solution, subculture, and its field control effect, so as to quickly transform the new strain into a microbial insecticide and apply it to production practice.

## Conclusion

The results derived from the study on the pathogenicity assessment of four entomopathogenic fungal strains strongly indicate that the isolate SG-A of *M. pingshaense* has the most effective pathogenicity against whiteflies and aphids and can be used as an excellent candidate to control both insect pests. The screening and assessment of four *M. pingshaense* isolates under laboratory conditions not only provide an important theoretical basis for the subsequent prevention and control of whiteflies and aphids for facility vegetable cultivations, but also shed some light for further research and development on establishing their fermentation protocol and application.

## Supporting information

S1 Fig Overall situation of *M. pinghaense* infestation of aphids. (TIF)

S2 Fig Overall situation of *M. pinghaense* infestation of whiteflies. (TIF)

S1 Data Original Data. (XLSX)
