# Supplementary figures and images for: In vitro isolation and identification of entomopathogenic fungus (Metarhizium pinghaense) and assessment of its virulence against whiteflies and aphids
Source: PLoS One. 2026 Jul 22;21(7):e0338072. doi: 10.1371/journal.pone.0338072 (PMC13390835; doi:10.1371/journal.pone.0338072)

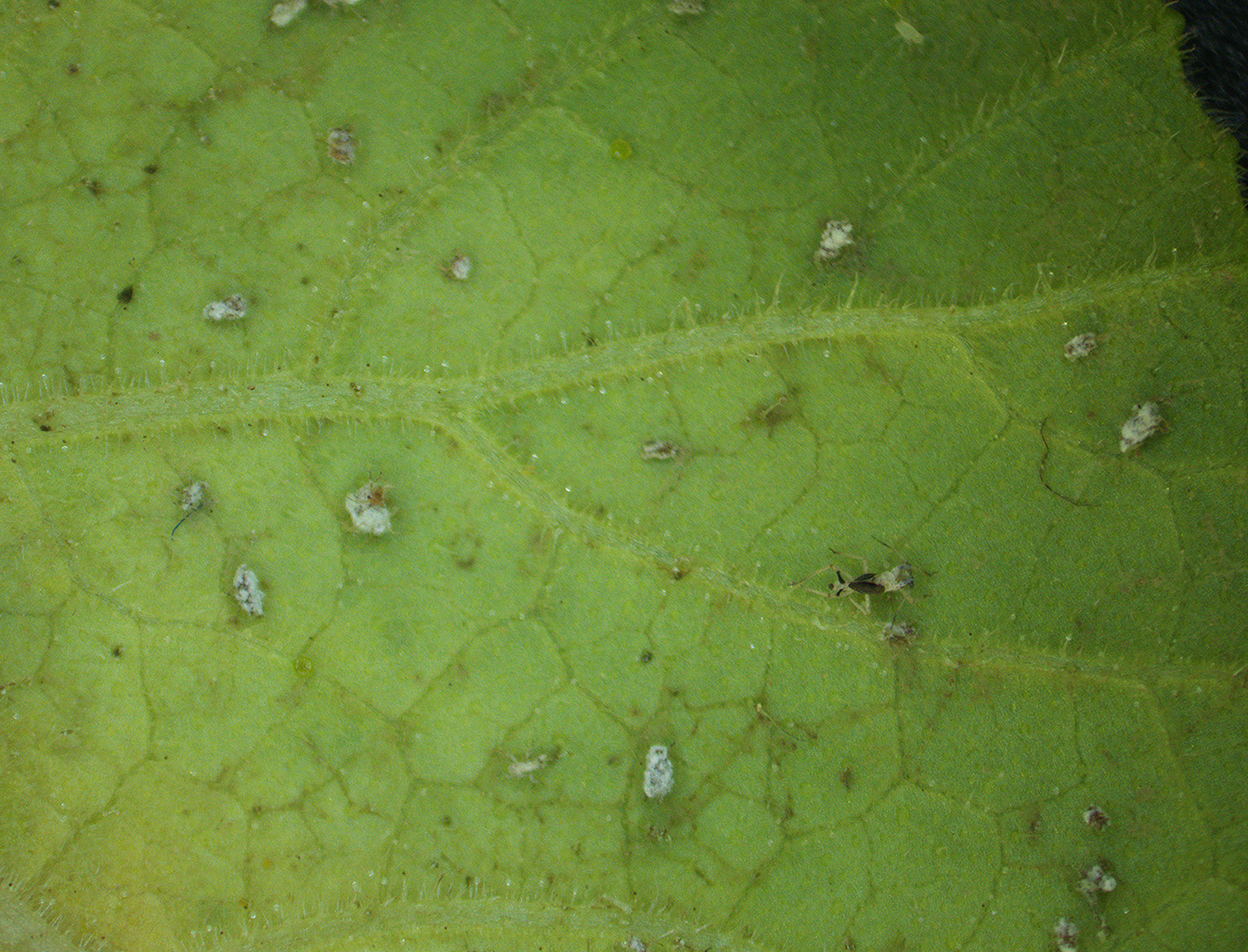

Supplement: S1 Fig — (TIF) [file pone.0338072.s001.tif]

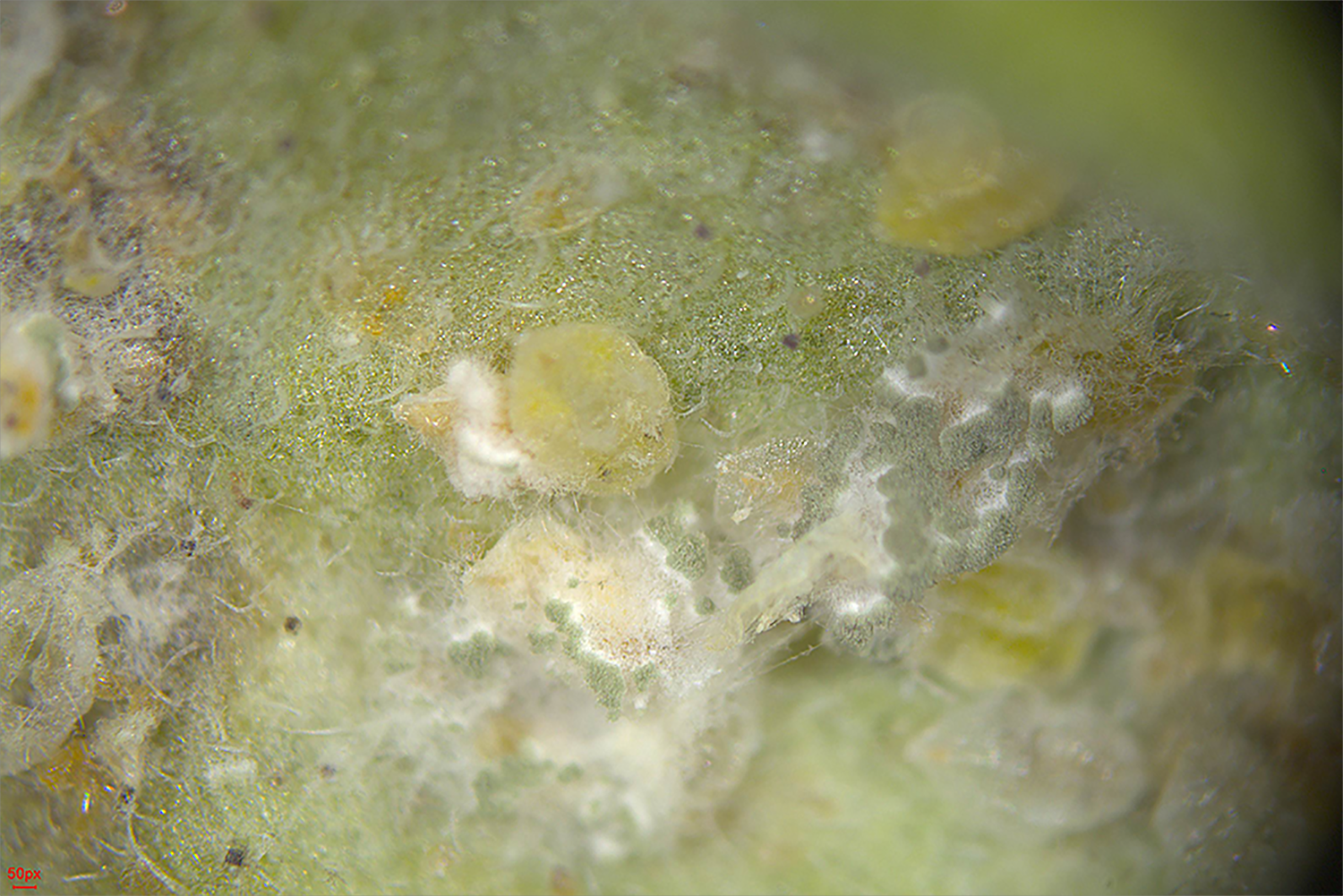

Supplement: S2 Fig — (TIF) [file pone.0338072.s002.tif]
